# The inhibition of the MRN complex by Mirin radiosensitizes particularly HPV-negative HNSCC cell lines

**DOI:** 10.1186/s12935-026-04218-1

**Published:** 2026-02-04

**Authors:** Laura S. Hildebrand, Babak Pornour Mehravani, Mohammed Khalifa, Paula Schiller, Janis Langkrär, Tina Jost, Rainer Fietkau, Luitpold V. Distel

**Affiliations:** 1 Department of Radiation Oncology, Universitätsklinikum Erlangen, Friedrich-Alexander-Universität Erlangen-Nürnberg (FAU), Universitätsstraße 27, 91054 Erlangen, Germany; 2 Comprehensive Cancer Center Erlangen-EMN (CCC ER-EMN), Erlangen, Germany; 3 Department of Radiation Oncology, Translational Radiobiology, Universitätsklinikum Erlangen, Friedrich-Alexander-Universität Erlangen-Nürnberg (FAU), Universitätsstraße 27, 91054 Erlangen, Germany

**Keywords:** Head and neck squamous cell carcinoma, Human papilloma virus, DNA damage response, Small molecule inhibitor, MRN complex, Mirin, Radiosensitization, Radiotherapy

## Abstract

**Background:**

Head and neck squamous cell carcinoma (HNSCC) is a relevant cancer entity with two main risk factors. Human papilloma virus (HPV)-positive ones are induced by virus infection and generally have a good prognosis due to their chemo- and radiosensitivity. In contrast, HPV-negative HNSCCs are primarily caused by tobacco and alcohol abuse; patients have a poor prognosis resulting in the need of innovative targeted and combinatory treatment options. Therefore, we combined the Mre11-Rad50-Nbs1 (MRN) inhibitor Mirin with ionizing radiation (IR). Our hypothesis is that the inhibition of the cancer cells’ DNA damage response (DDR) by Mirin leads to reduced repair capacity and a radiosensitization of the cancer cells.

**Methods:**

We investigated the effect of Mirin in combination with IR on five HPV-negative and two HPV-positive HNSCC cell lines and one primary fibroblast cell line - serving as healthy control - in several functional assays.

**Results:**

We suggest – on the one hand - that Mirin shows a trend towards radiosensitizing effects regarding cell death, cell cycle distribution, colony formation, and deoxyribonucleic acid (DNA) damage in HPV-negative HNSCC cell lines but not in HPV-positive ones. On the other hand, the healthy control was nearly unaffected by the combinatory treatment which indicates low side effects.

**Conclusions:**

It is useful to generate a deeper insight into the underlying cellular mechanisms of Mirin response in future studies and further validate Mirin’s potential radiosensitizing effect in HPV-negative HNSCCs.

**Graphical abstract:**

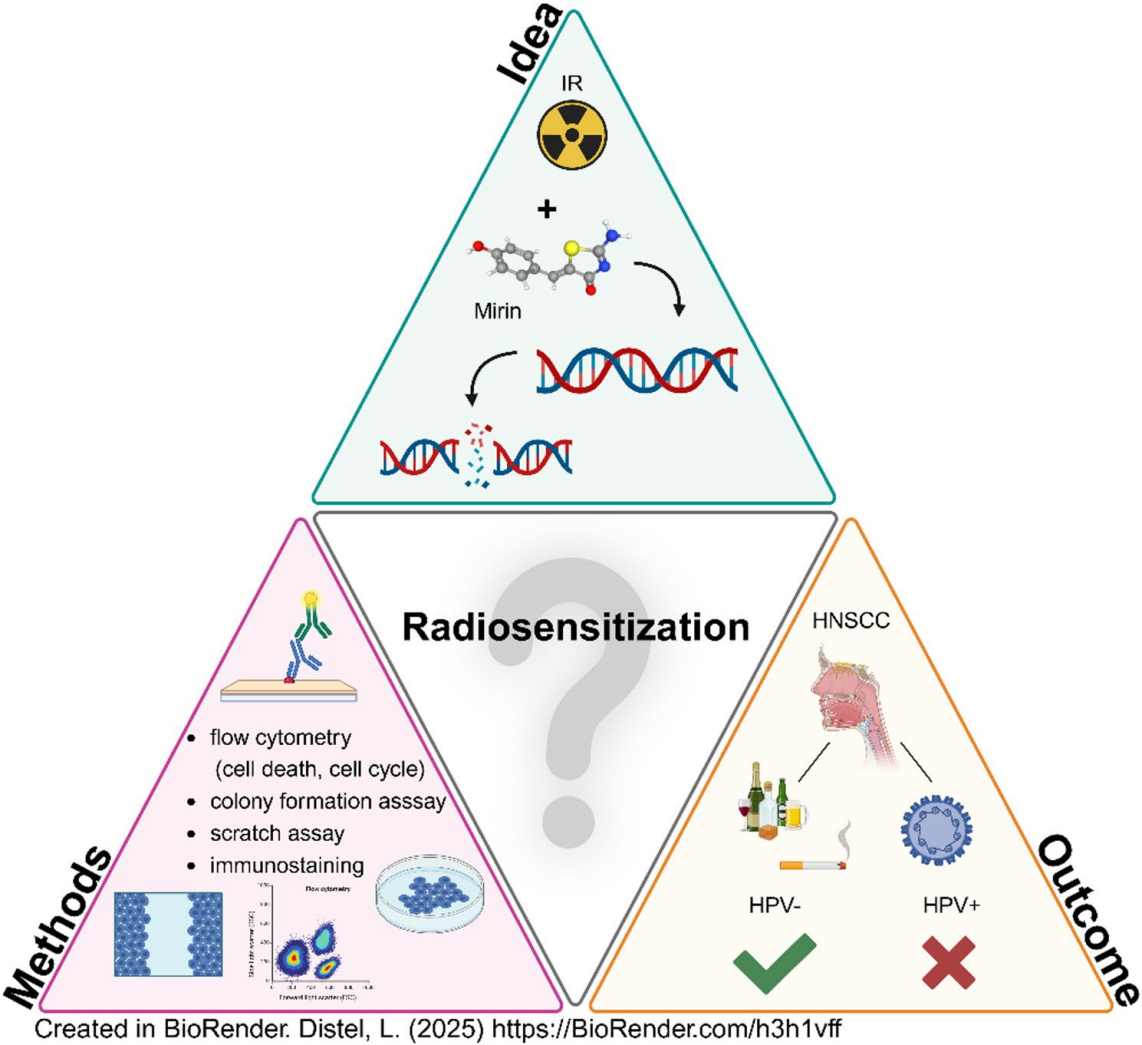

**Supplementary Information:**

The online version contains supplementary material available at 10.1186/s12935-026-04218-1.

## Background

Head and neck squamous cell carcinoma (HNSCC) is a heterogenous disease with two major risk factors: on the one hand, tobacco and alcohol abuse; on the other hand, the cancer induction by human papilloma virus (HPV) [1]. The two subgroups are differing in patient characteristics and clinical outcome; generally speaking, HPV-positive HNSCCs are more responsive to chemo- and radiotherapy and therefore patients have a better prognosis [2–4]. Standard of care are surgery, chemotherapy, and radiotherapy [5]. Nevertheless, advanced treatment options are urgently needed; especially for patients with prognostically poor HPV-negative tumors [1, 5]. Our focus is on finding promising combinatory treatments for radiotherapy.

In healthy cells, DNA damage response (DDR) is crucial for maintaining genomic stability and avoiding persistent mutations [6]. After an endogenous or exogenous deoxyribonucleic acid (DNA) damage, cells either repair their DNA correctly or inactivate themselves to avoid the spread of mutated cells. In contrast, DDR is often impaired in cancer cells, which prevents sufficient repair or inactivation and therefore promoting cancerogenesis [6]. Ionizing radiation (IR) used in radiotherapy induces double strand breaks (DSBs), which should lead to cell death but is often not sufficient in cancer cells [7, 8]. Treatment response is thereby correlated with the cancer cells’ ability to process DNA damage [7]. It is known that a high DNA repair capacity is associated with therapy resistance [7, 9]. Accordingly, defects in DDR may sensitize cancer cells to DNA-damaging therapies [6]. Evaluating effective and safe combinations of cancer treatments is a common approach to improve patients’ prognosis and quality of life [10], including the combination of IR with small molecule inhibitors (SMI) of the DDR [11]. There is already broad preclinical and clinical evidence that artificially inhibiting DDR with a SMI is a potential combinatory strategy [12] to radiosensitize head and neck cancer and improve their clinical outcome; for example, the DNA-dependent protein kinase inhibitor Peposertib was already tested in preclinical HNSCC model systems [13] and in a phase 1 trial in combination with IR in patients with HNSCCs [14].

In mammalian cells it is essential that the DDR is activated immediately after a DNA damage occurs. One key regulator in early DDR is the Mre11-Rad50-Nbs1 (MRN) complex - consisting of MRE11 homolog, double strand break repair nuclease (Mre11), Rad50 double strand break repair protein (Rad50), and nibrin (Nbs1) - which has sensing and repair functions [15]. The MRN complex is recruited to the DNA DSB seconds after it occurred and is responsible for the activation of ataxia telangiectasia mutated (ATM) by its autophosphorylation at serine (Ser)1981 [6, 15–17]. ATM induces a downstream signal cascade that leads to cell cycle arrest, DNA repair, or cell death ensuring genome integrity [6, 15]. Healthy cells have multiple DNA repair pathways available but IR-induced DNA damage is repaired primarily by non-homologous end joining (NHEJ) and homologous recombination (HR) [6, 18]. Pathway choice is principally dependent on the cell cycle phase of the damaged cell [16, 19]; but the endonuclease activity of MRN allows the generation of 3’ single-stranded DNA overhangs necessary for HR and drives the cell to HR instead of NHEJ [15, 17, 20]. Even if the MRN complex is mostly associated with HR [17, 18], it also plays a role in tethering DNA for NHEJ and the choice of the DNA DSB repair pathway [15, 16, 19]. For both pathways Rad50 is relevant for DNA binding [15].

Already in 2008, Dupré et al. presented the SMI Mirin targeting the MRN complex [21]. They evaluated Mirin’s mode of action in depth and stated that it inhibits the MRN complex – more detailed Mre11 – and eventually MRN-dependent ATM activation resulting in impaired DDR after DNA DSBs, while the ATM activity itself and MRN-independent ATM activation are not impaired by Mirin [21]. Mirin does not destabilize the MRN complex but it inhibits the Mre11 3’ to 5’ exonuclease activity [21]. Additionally, Shibata et al. carried out structural analysis of the interaction of Mirin and Mre11 concluding that Mirin does not directly bind to the nuclease site of Mre11 but prevents the DNA from binding and therefore inhibits the opening of double-stranded DNA and the exonuclease activity of Mre11 [22]. However, Aasumets et al. recently showed that Mirin’s effect is not limited to Mre11 inhibition but influences also immune response and mitochondrial DNA [23].

According to Alblihy et al. [24, 25], ovarian cancer patients with low BRCA2 DNA repair associated (BRCA2) expression concomitant with high Mre11 expression have a bad prognosis, which implies that Mre11 inhibition could be of clinical relevance in ovarian cancer. Even if Mirin’s potential to improve treatment of cancer cells was investigated in several studies including different entities, for example neuroblastoma [26] and ovarian cancer [24, 27], yet the knowledge concerning HNSCCs is limited. Wang et al. described the radiosensitizing effect of Mirin in two esophageal squamous cell cancer cell lines [28] but there are no studies available including a broader variety of HNSCC cell lines classified according to their HPV-status. In our study, we want to answer the question if Mirin may serve as a potential radiosensitizer for HNSCCs, especially the HPV-negative ones.

## Methods

### Cell cultivation

We used a panel of seven HNSCC cell lines and one healthy fibroblast cell line to determine the effect of the combined treatment of Mirin and IR on HPV-positive (UD-SCC-2, UM-SCC-47) and HPV-negative (Cal33, CLS-354, Detroit 562, HSC4, RPMI 2650) HNSCCs compared to healthy cells (SBLF24). Fibroblasts were chosen as healthy control as the skin is always affected by radiotherapy and skin toxicity is common. All cell lines were cultivated in Dulbecco’s modified eagle medium (PAN-Biotech GmbH, Aidenbach, Germany) with 10 % fetal bovine serum (FBS) (FBS Superior, Sigma-Aldrich, Darmstadt, Germany) and 1 % penicillin-streptomycin (Gibco, Waltham, USA). Cells were incubated at 37 °C and 5 % CO_2_ in a humidified atmosphere and split (0.5 % trypsin-ethylenediaminetetraacetic acid, Gibco, Waltham, USA) every three to four days in an appropriate ratio to avoid complete confluence. Skin fibroblasts named SBLF24 were obtained by biopsy from the forearm of a 28-year-old male healthy donor. Isolation and cultivation of fibroblasts from skin biopsy were described elsewhere [29]. The procedure was approved by the Ethics Committee of the medical faculty of Friedrich-Alexander-Universität Erlangen-Nürnberg (204_17 BC) on August 18, 2017 and the donor signed the informed consent. The other cell lines are commercially available. CLS-354, Detroit 562, and RPMI 2650 were bought from CLS Cell Lines Service GmbH (Eppelheim, Germany). Dr. Thorsten Rieckmann (University Medical Centre Hamburg-Eppendorf, Germany) kindly provided Cal33, HSC4, UD-SCC-2, and UM-SCC-47. Mycoplasma testing was done on a regularly basis using Mycoplasma Detection Kit (InvivoGen Europe, Toulouse, France).

### Treatment

**Fig. 1:**
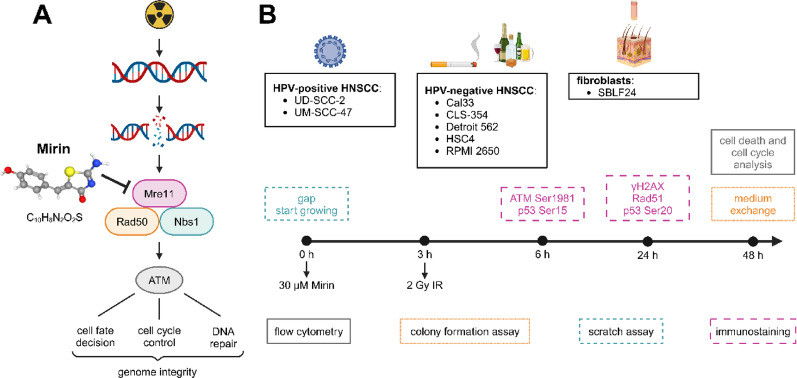
Overview of Mirin’s target structure in DNA damage response (DDR) and the applied experimental setup. (**A**) Scheme of induction of deoxyribonucleic acid (DNA) double strand breaks (DSBs) by ionizing radiation (IR), response of the Mre11-Rad50-Nbs1 (MRN) complex - consisting of MRE11 homolog, double strand break repair nuclease (Mre11), Rad50 double strand break repair protein (Rad50), and nibrin (Nbs1) - and ataxia telangiectasia mutated (ATM), and resulting cellular response with the aim to maintain genome integrity. Mirin inhibits the MRN complex by targeting Mre11. (**B**) Overview of the used cell lines, analytical techniques, and the experimental timeline. We studied human papilloma virus (HPV)-positive and HPV-negative head and neck squamous cell carcinoma (HNSCC) cell lines and primary skin fibroblasts. After an appropriate incubation time following cell seeding, cells were treated with 30 µM Mirin and three hours afterwards with a single dose of 2 Gy (Gy) IR. Treatment time was dependent on the assay. Adapted, [6, 15, 21, 30]. Created in BioRender. Distel, L. (2025) https://BioRender.com/o91b145

Cells were treated with a combination of 30 µM Mirin and 2 Gy (Gy) IR. Therefore, a working solution with 10 mM Mirin (catalogue number S8096, molecular weight 220.25, Selleck Chemicals LLC, Houston, USA) in dimethyl sulfoxide (DMSO) (Carl Roth GmbH + Co. KG, Karlsruhe, Germany) was prepared and pipetted directly into the cultivation medium to reach the final concentration (Mirin); the working solution was stored at −80 °C and thawed freshly for every experiment. To ensure the uptake of Mirin into the cells, three hours of incubation followed. Three hours after Mirin addition, a part of the cultivation vessels was additionally irradiated with 2 Gy IR (Mirin + IR) with an ISOVOLT Titan X-ray generator (GE Inspection Technologies, Ahrensburg, Germany; X-ray tube, 2 Gy/min, 120 kV, 2 mm aluminium filter). The dose of 2 Gy IR was chosen as a representative single dose of a normo-fractionated irradiation scheme in HNSCC. As controls served cells only treated with DMSO corresponding to the used volume of the Mirin working solution (Co) and cells that only received 2 Gy IR (IR) respectively. For immunostaining, DMSO control was replaced by completely untreated cells (Fig. 1).

### Flow cytometry

For cell death and cell cycle analysis, an appropriate number of cells was seeded in cell culture flasks to reach 80 % confluence after three days. After 24 h of incubation, medium was changed to cultivation medium with reduced FBS amount (2 %) and treatment was carried out as described above immediately. 48 h after Mirin treatment, cells were harvested. Therefore, flasks were washed with Dulbecco’s phosphate buffered saline (DPBS) (Gibco, Waltham, USA), cells were detached with trypsin, and trypsin reaction was stopped with 2 % cultivation medium. Supernatant, DPBS, and cell suspension of every condition were collected and centrifuged. Cell pellet was resuspended and divided in two equal parts for cell death and cell cycle analysis.

#### Cell death analysis

Subsequent to harvesting, 200 µL cold Ringer solution (Fresenius Kabi AG, Bad Homburg, Germany) and allophycocyanin (APC) Annexin V/7-Aminoactinomycin D (7AAD) (BioLegend, Inc., San Diego, USA/BD Biosciences, Franklin Lakes, USA) mix (1:1) were added to the cells. An incubation of 30 min at 4 °C in the dark and afterwards centrifugation followed. Pellets were resuspended and another 150 µL Ringer solution were added before measuring the forward and sideward scatter light, Annexin V, and 7AAD signal of the stained cell suspension at the Cytoflex S (Beckman Coulter GmbH, Krefeld, Germany).

#### Cell cycle analysis

For fixation, 1 mL 2 % cultivation medium and 10 mL cold 70 % ethanol (Otto Fischar GmbH & Co. KG, Saarbrücken, Germany) were added to the cells and an incubation at 4 °C at least overnight followed. Afterwards, tubes were centrifuged and pellets were resuspended before the addition of 1 mL cold Ringer solution and 3 µL Hoechst 33342 (10 mg/mL, Molecular Probes, Waltham, USA) per sample. After one hour of incubation at 4 °C in the dark, cells were centrifuged and pellets were resuspended in 150 µL Ringer solution followed by measurement of forward and sideward scatter light and Hoechst signal at the Cytoflex S.

### Colony formation assay

The colony formation assay was used to determine the cells’ clonogenicity after treatment. Cells were seeded in Petri dishes using a cell line- and condition-dependent cell number (300–1200 cells/Petri dish) that ensures separation of individual cells and the individual colonies during cultivation. Cells were incubated overnight and treatment followed before the first cell division as described above. 48 h after Mirin addition, medium was exchanged to standard cultivation medium without Mirin or DMSO. The following incubation period varied from seven to 14 days depending on the cell line. When colonies of at least 50 cells were detectable under the microscope in all conditions, medium was removed and colonies were stained with Wright’s eosin methylene blue solution (Carl Roth GmbH + Co. KG, Karlsruhe, Germany) for 45 min. Methylene blue was discarded and remaining dye was washed away with water. After drying, visible colonies were counted manually or with Biomas Software (V3.0 7/2012, Erlangen, Germany). Plating efficiency (PE) was calculated by dividing counted colonies by the seeded cell number. Surviving fraction (SF) is defined as the proportion of the PE of treated conditions to the PE of the DMSO control.

### Scratch assay

For the scratch assay the life-cell-imaging system zenCELL owl (innoMe GmbH, Espelkamp, Germany) with two-well culture-inserts (ibidi GmbH, Gräfelfing, Germany) to create a defined gap were used. One two-well silicone insert was placed in every well of a 24-well cell culture plate and 50,000 cells were seeded into each well of the two-well culture-inserts to reach complete confluency after 30–36 h of incubation. Inserts were removed, which creates a clear gap between the cell layers in the two wells. Immediately, fresh cultivation medium was added and treatment was done as described above. After irradiation, the plate was placed in the life-cell-imaging microscope to detect the gaps. Once an hour an image was automatically acquired by the system until the gap was completely closed in all conditions.

Each experiment including all four conditions (DMSO, IR, Mirin, Mirin + IR) was analyzed when the scratch in the DMSO control was completely closed (A_DMSO_ = 0 mm²). The residual scratch area in the treated conditions at this time point (A_condition_) was calculated using Biomas Software (V3.0 7/2012, Erlangen, Germany). The initial scratch at three hours was 0.6 mm² (A_initial_). The proportion of the remaining scratch from the initial scratch was calculated (see formula 1) and plotted.

1 $$\:Residual\:scratch\:area=\:\frac{{A}_{condition\:}{[mm}^{2}]}{{A}_{initial}\:{[mm}^{2}]}$$

### Immunostaining

Cells were seeded directly onto glass slides or into eight-well silicone chambers (flexiPERM^®^, SARSTEDT AG & Co. KG, Nümbrecht, Germany) on the glass slides and were incubated 48 h before the treatment was carried out as described above. Slides used for staining γH2A.X variant histone (H2AX), Rad51 recombinase (Rad51), and tumor protein P53 (p53) Ser20 were incubated 21 h after irradiation before fixation, whereas slides used for staining p53 Ser15 and ATM Ser1981 were incubated only three hours after irradiation before fixation. We used different time points to consider the time-dependent cellular reaction to DNA damage. ATM Ser1981 [31] and p53 Ser15 [32, 33] are phosphorylated rapidly after a DNA damage. Also, p53 Ser20 can be phosphorylated quickly but it can be also detected at a later time point [33, 34]. We used γH2AX as a marker for DNA damage [35] and Rad51 as a marker for HR [36]; as we wanted to detect especially damages that persist longer and cannot be repaired rapidly, we measured those markers later. After the target specific repair time, slides were washed once with DPBS. For fixation and permeabilization, 4 %/0.1 % formaldehyde/Triton X-100 in DPBS (formaldehyde solution 37 %, Carl Roth GmbH + Co. KG, Karlsruhe, Germany/Triton™ X-100, Sigma-Aldrich, Darmstadt, Germany) was added to the slides for 15 min. Afterwards, a five-minute washing with 1x Tris-buffered saline (TBS) was carried out three times. Slides were incubated in blocking solution (1 % bovine serum albumin [BSA] [SERVA Electrophoresis GmbH, Heidelberg, Germany], 10 % FBS in DPBS) at least overnight at 4 °C. Before continuing, slides were again washed three times in TBS. Primary and secondary antibodies were diluted in 1 % BSA in TBS (Table 1). Primary antibody mix was added to the slides and incubated overnight at 4 °C in a humidified atmosphere. The next day, three washing steps with TBS followed before the secondary antibodies were incubated 90 min at room temperature in the dark. Finally, slides were washed again three times with TBS and once with distilled water. After drying, slides were mounted with Vectashield^®^ Plus antifade mounting medium with 2-(4-Amidinophenyl)−1*H*-indole-6-carboxamidine (DAPI) (Vector Laboratories, Inc., Burlingame, USA) and coverslips. Microscopic images were acquired using Axio Imager.Z2 fluorescence microscope (Carl Zeiss AG, Oberkochen, Germany; 630x magnification) with imaging software Metafer 4 (V 3.10.7, MetaSystems Hard and Software GmbH, Altlussheim, Germany) and foci per nucleus were counted using Biomas Software (V3.0 7/2012, Erlangen, Germany) considering the focus signal proportional to the background. For every biological replicate, an assembled image of 7 × 7 single images was acquired in an area where cells were dense and every nucleus was analyzed.

**Table 1:** Primary and corresponding secondary antibodies used for immunostaining

Target	Primary antibody	Secondary antibody
Rad51	RAD51; catalogue number: sc-56,212; host: mouse; dilution: 1:50 (Santa Cruz Biotechnology, Inc., Dallas, USA)	Donkey anti-Mouse IgG (H + L) Highly Cross-Adsorbed Secondary Antibody, Alexa Fluor™ Plus 555; catalogue number: A-32,773; host: donkey; dilution: 1:200 (Invitrogen, Thermo Fisher Scientific Inc., Waltham, USA)
ATM Ser1981	Phospho-ATM (Ser1981) (10H11.E12) Mouse mAb; catalogue number: 4526; host: mouse; dilution: 1:300 (Cell Signaling Technology, Inc., Danvers, USA)	Donkey anti-Rabbit IgG (H + L) Highly Cross-Adsorbed Secondary Antibody, Alexa Fluor™ 488; catalogue number: A-21,206; host: donkey; dilution: 1:200 (Invitrogen, Thermo Fisher Scientific Inc., Waltham, USA)
p53 Ser15	Phospho-p53 (Ser15) (16G8) Mouse mAb; catalogue number: 9286; host: mouse; dilution: 1:100 (Cell Signaling Technology, Inc., Danvers, USA)	
γH2AX	Phospho-Histone H2A.X (Ser 139) (20E3) Rabbit mAb; catalogue number: 9718; host: rabbit; dilution: 1:400 (Cell Signaling Technology, Inc., Danvers, USA)	
p53 Ser20	Phospho-p53 (Ser20) Antibody; catalogue number: 9287; host: rabbit; dilution: 1:100 (Cell Signaling Technology, Inc., Danvers, USA)	

### Software

Raw data analysis of flow cytometry data was done using Kaluza Analysis Version 2.3.00000.20268. Image acquisition for the scratch assay was carried out with zenCELL owl Version v3.4.1. Schematic images were generated with BioRender (Science Suite Inc., Toronto, Canada). Immunostaining was presented and analyzed with Biomas Software (V3.0 7/2012, Erlangen, Germany). For processing of raw data Microsoft Excel 2019 (Microsoft Corporation, Redmond, USA), for presenting data and statistical analysis GraphPad Prism 9.5.1 (GraphPad Software, San Diego, USA) were used. For statistical analysis, biological replicates of the assays were carried out. For all assays except of immunostaining, the number of biological replicates was at least four; the exact number is indicated as n in the figure legend. Results generated with less repetitions are indicated in the results part and can only be interpreted as tendencies. Due to the number of biological replicates, it could not be tested if the results are normally distributed; this is why we used the non-parametric two-tailed Mann-Whitney test (Fig. 6 one-tailed due to limited replication number). There were no multiple testing corrections but we always compared IR with Mirin + IR to evaluate the radiosensitizing effect of Mirin.

## Results

We hypothesize that inhibiting DDR in combination with IR leads to a radiosensitization of cancer cells because of the artificially reduced ability to repair the IR-induced DNA damage. To improve the prognosis of HNSCCs - especially of the radioresistant HPV-negative ones –, we combined the MRN inhibitor Mirin with IR and investigated its radiosensitizing effect on HPV-positive and HPV-negative HNSCC cell lines and healthy fibroblasts by various functional assays.

### Combination of Mirin and IR induces more cell death than IR alone in HPV-negative HNSCCs

**Fig. 2:**
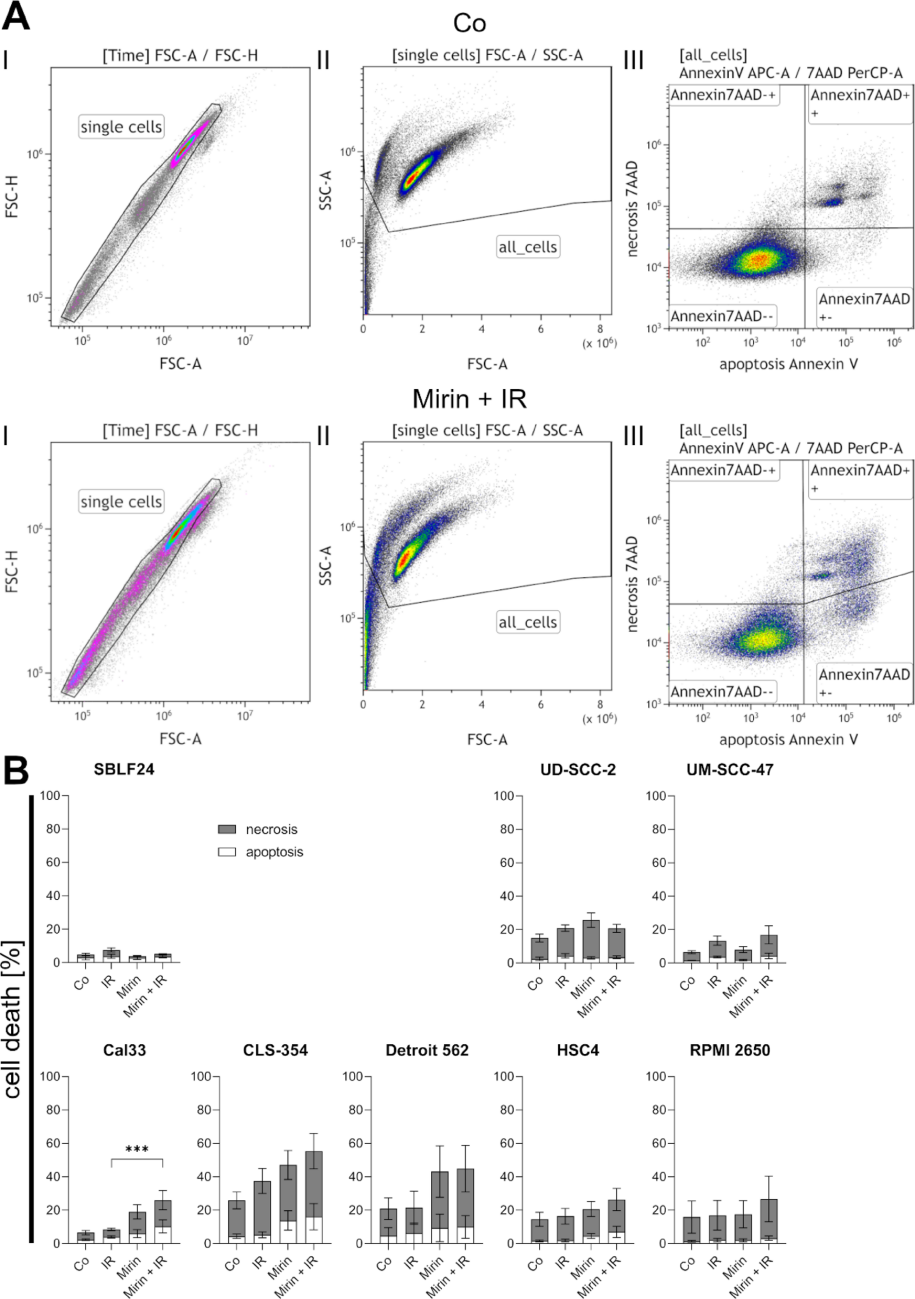
Cell death analysis with flow cytometry. (**A**) Gating strategy: Doublets (I) and cell debris (II) were excluded. Annexin/7-Aminoactinomycin D-negative (Annexin7AAD–) cells were counted as living, Annexin7AAD+- as apoptotic, and Annexin7AAD + + as necrotic (III). Cal33 treated with a combination of Mirin and IR or a vehicle control (Co) are shown exemplary. (**B**) Amount of dead cells (apoptotic: white, necrotic: grey) in HNSCC cell lines and healthy fibroblasts (SBLF24). Cells were treated with 30 µM Mirin and three hours afterwards additionally with a single dose of 2 Gy IR (Mirin + IR), only 30 µM Mirin (Mirin), or only 2 Gy IR (IR). The same volume dimethyl sulfoxide (DMSO) as used for 30 µM Mirin served as vehicle control (Co). Statistically significant differences between IR and Mirin + IR (sum of apoptotic and necrotic cells) were calculated with two-tailed Mann-Whitney test, *** *p* = 0.0002. Each value represents mean ± standard deviation (SD) (*n* ≥ 4)

We investigated the induction of cell death by 30 µM Mirin in combination with 2 Gy IR in our cell line panel by flow cytometry. We opted for 30 µM because cell death was detectable in pilot experiments in Cal33 and HSC4, but only limited cell death occurred, roughly corresponding to the effect of a dose of 2 Gy IR, leaving enough viable cells to observe an effect when IR and Mirin were combined. We would expect a potentiation of the effect in a fractionated scheme; that’s why we want to avoid excessive cell killing with a single dose of 2 Gy IR (compare to Supplementary Fig. 1). Forward and side scatter allowed exclusion of doublets (Fig. 2, A, I) and cell debris (Fig. 2, A, II) whereas Annexin V and 7AAD allowed discrimination between living, apoptotic and necrotic cells (Fig. 2, A, III). Annexin V-positive cells were counted as apoptotic (Annexin7AAD+-), double positive ones as necrotic (Annexin7AAD++), and double negative ones as living (Annexin7AAD–). In Cal33, the combined treatment of Mirin + IR significantly (*p* = 0.0002) elevated the amount of dead cells compared to irradiation alone, which indicates a radiosensitizing effect of Mirin in this cell line. In all the other cell lines, there were no significant differences between IR and Mirin + IR but some tendencies could be observed: In the healthy fibroblasts (SBLF24) IR slightly induced cell death but neither Mirin alone nor the combination of Mirin with IR induced additional cell death compared to the control. Same is true for the HPV-positive cell lines UD-SCC-2 and UM-SCC-47, where cell death was slightly elevated by IR but there was no additional effect by Mirin. In contrast, all HPV-negative cell lines had the tendency to respond to the combined treatment, even if it was only statistically significant in Cal33. In Cal33, CLS-354, and Detroit 562 Mirin alone already induced cell death compared to control. Additionally, in all cell lines the amount of dead cells was higher in the combined treatment than in IR alone, which points to a potential radiosensitization of the HPV-negative HNSCCs by Mirin. Generally, in all cell lines the effects were mainly based on induction of necrosis whereas apoptosis was slightly influenced (Fig. 2, B).

### Mirin in combination with IR induces G2/M arrest in HPV-negative HNSCCs

**Fig. 3:**
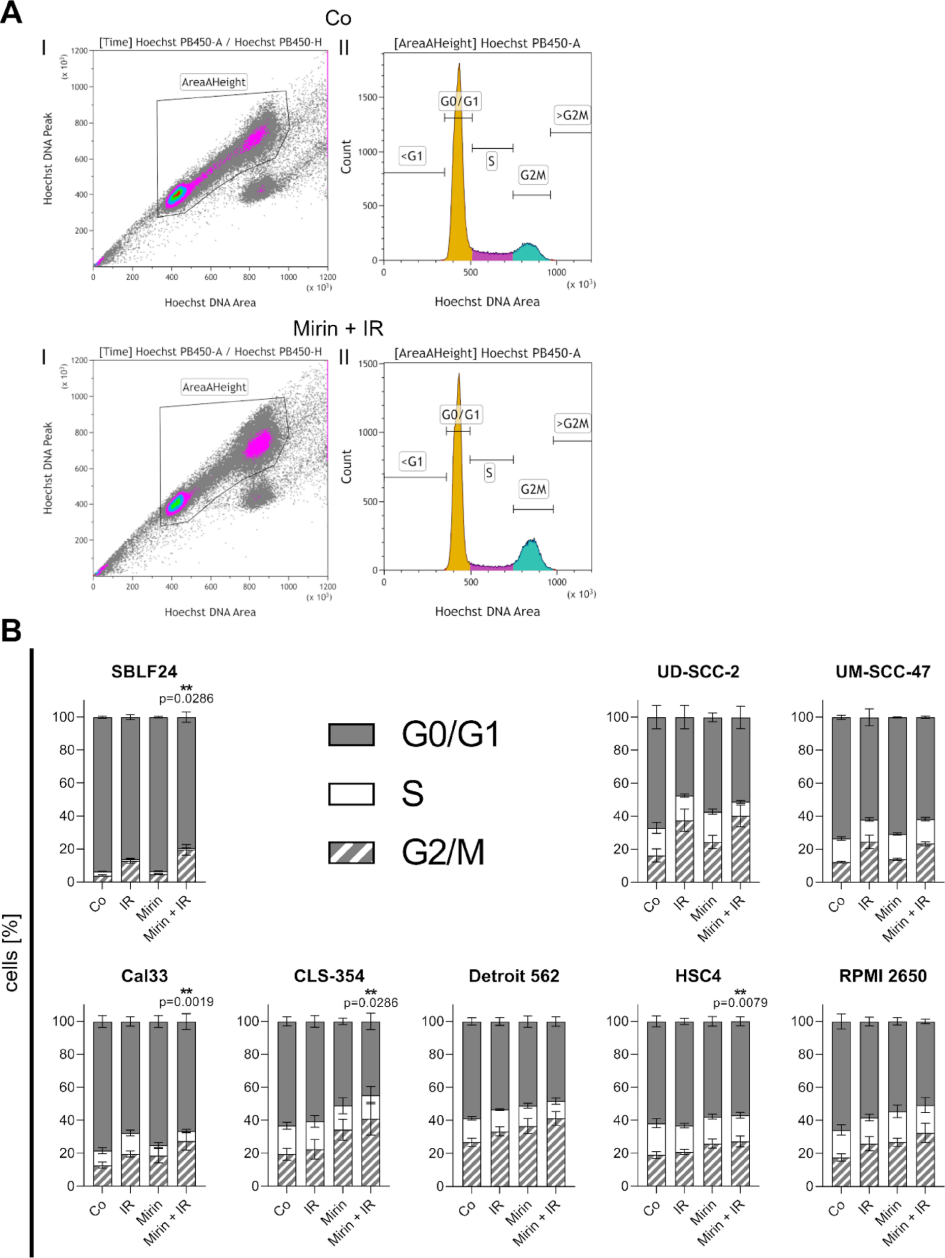
Cell cycle analysis with flow cytometry. (**A**) Gating strategy: Doublets and cell debris were excluded (I). Cell cycle phases were discriminated according to the cells’ DNA content (II). Cal33 treated with a combination of Mirin and IR or a vehicle control (Co) are shown exemplary. (**B**) Cell cycle distribution (G0/G1: grey, S: white, G2/M: dashed) of HNSCC cell lines and healthy fibroblasts (SBLF24). Cells were treated with 30 µM Mirin and three hours afterwards additionally with a single dose of 2 Gy IR (Mirin + IR), only 30 µM Mirin (Mirin), or only 2 Gy IR (IR). The same volume DMSO as used for 30 µM Mirin served as vehicle control (Co). Statistically significant differences between IR and Mirin + IR (G2/M) were calculated with two-tailed Mann-Whitney test. Each value represents mean ± SD (*n* ≥ 4)

As radiosensitivity of cells differs between the different cell cycle phases with a peak in the G2/M phase, we investigated the cell cycle distribution of the cell line panel after treatment with flow cytometry. We stained DNA with Hoechst, which allows us to determine the DNA content of the cells dependent on their cell cycle phase. At first, doublets and cell debris were excluded (Fig. 3, A, I). Secondly, the amount of cells in the single cell cycle phases was determined according to their DNA amount corresponding with the Hoechst signal (Fig. 3, A, II). The G2/M fraction in SBLF24 was significantly increased by Mirin + IR compared to IR alone. In the HPV-positive cell lines UD-SCC-2 and UM-SCC-47, there was no statistically significant difference in the amount of cells in the G2/M phase after irradiation alone compared to the combined treatment with Mirin. As already shown for cell death analysis, also cell cycle distribution of HPV-negative cell lines was clearly different from HPV-positive ones. In Cal33, CLS-354, and HSC4 the combined treatment significantly increased the amount of cells in G2/M phase compared to IR. In Detroit 562 and RPMI 2650 at least a slight increase was detectable, even if it was not statistically significant (Fig. 3, B).

### Mirin and the combined treatment with IR reduce the clonogenicity of HPV-negative HNSCC cell lines

**Fig. 4:**
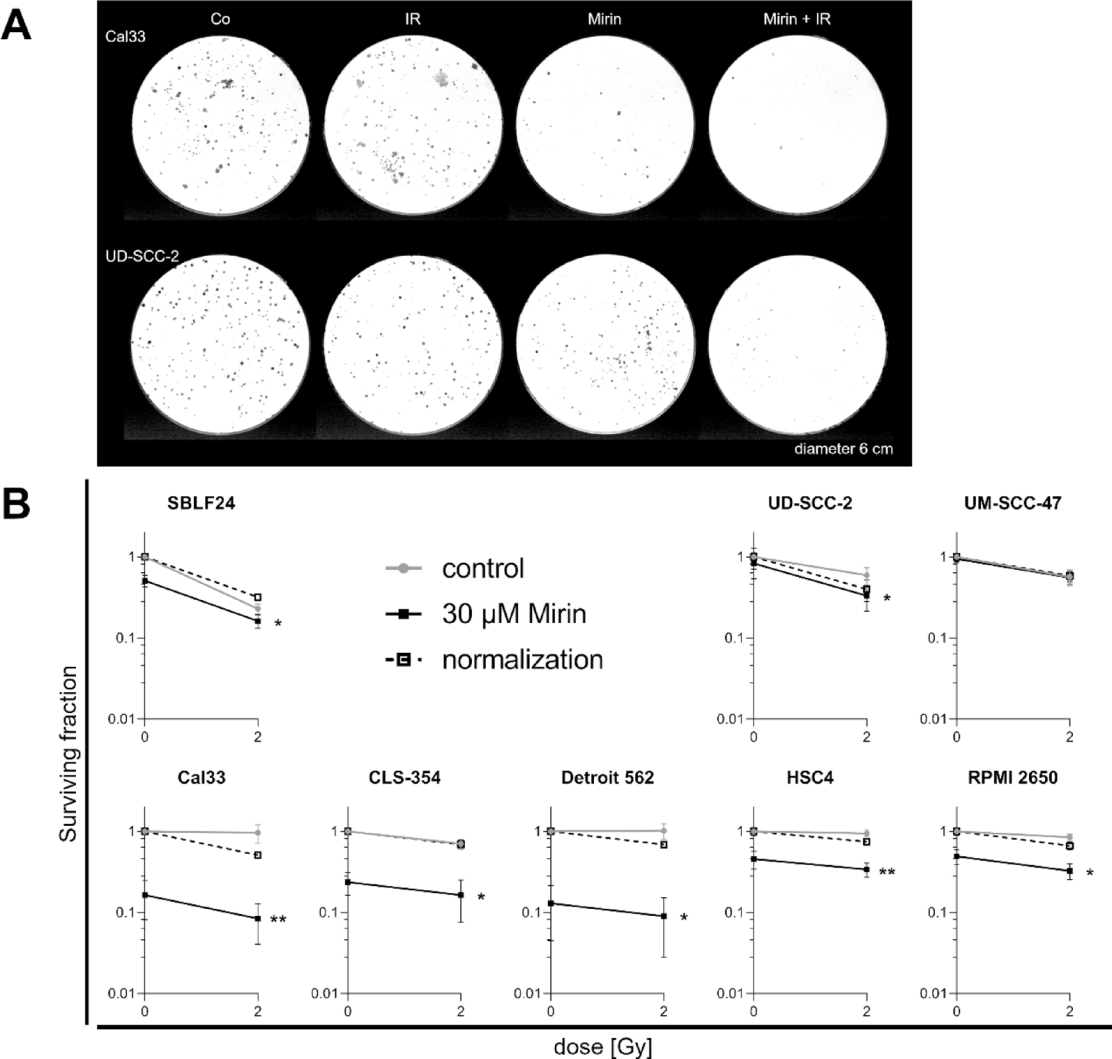
Colony formation assay. (**A**) Representative stained colonies after ten to 14 days of incubation: Cal33 and UD-SCC-2 treated with a vehicle control (Co), IR, 30 µM Mirin, or a combination of Mirin and IR are shown exemplary. (**B**) Surviving fraction (SF) of HNSCC cell lines and healthy fibroblasts (SBLF24) treated with 30 µM Mirin (black line) with or without additional 2 Gy IR after three hours. Cells that did not receive Mirin are represented by the grey line. DMSO according to the volume of Mirin served as vehicle control and the SF was calculated relative to it. For normalization, the Mirin line was shifted parallel into the starting point SF = 1 for easier comparison of the control and the Mirin line. Statistically significant differences between IR and Mirin + IR were calculated with two-tailed Mann-Whitney test, * *p* = 0.0286, ** *p* = 0.0079, except SBLF24 * *p* = 0.0108. Each value represents mean ± SD (*n* = 4 CLS-354, Detroit 562, RPMI 2650, UD-SCC-2; *n* = 5 Cal33, HSC4, UM-SCC-47; *n* = 6 SBLF24)

For colony formation assay, single cells were seeded into petri dishes, treated, and resulting colonies were stained and quantified. The SF relative to the control was calculated (Fig. 4, A). The clonogenicity of SBLF24 and the HPV-positive cell lines UD-SCC-2 and UM-SCC-47 was not or only slightly impaired by Mirin (black line, data point on the left), whereas the SF of HPV-negative cell lines was clearly reduced by Mirin. Comparing IR with Mirin + IR, the results are similar for HPV-positive and HPV-negative cell lines. The SF was significantly reduced in all cell lines except UM-SCC-47 by the combined treatment compared to IR alone. For better visualization of the difference between IR alone and the combined treatment, we did a normalization of the Mirin conditions. For that reason, we made a parallel shift of the line representing the Mirin conditions into the starting point (SF = 1) of the control line without Mirin. Because of the same starting point, the slope of the control line and the normalized line, which has the same slope as the Mirin line but a different starting point, can be compared easily. The steeper the slope, the stronger the effect of the treatment (grey line IR, dashed line Mirin + IR). If the normalized line is steeper and below the control line, we interpret this as radiosensitization. However, the original data are presented as the Mirin line (black) and are used for statistical analysis; the normalization is an additional information. In the normalized presentation it gets clear that for SBLF24, UM-SCC-47, and CLS-354 there was no additional effect of the combined treatment, but the effect represents only the sum of Mirin and IR. In contrast, in the other cell lines the combined treatment had an additional effect that is larger than only the sum of Mirin and IR (Fig. 4, B).

### The combined treatment reduced the migration ability of cancer cells

**Fig. 5:**
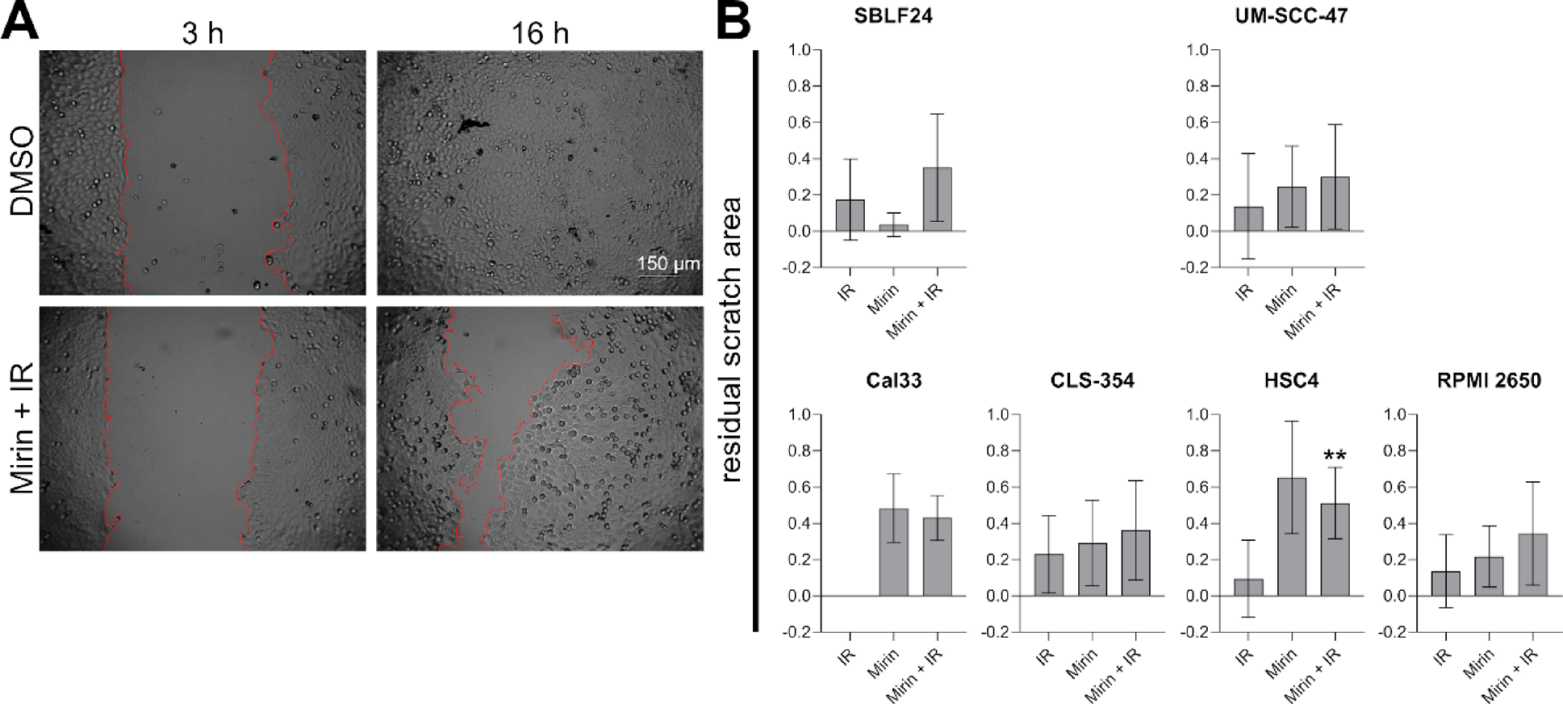
Scratch assay of HNSCC cell lines and healthy fibroblasts. (**A**) Representative images of Cal33. Cells were treated with 30 µM Mirin and three hours afterwards additionally with a single dose of 2 Gy IR (Mirin + IR) or a vehicle control (DMSO). The scratch area was determined and is indicated by the red line. The initial image (t = 3 h) and the time point when the scratch in the DMSO control was completely closed (t = 16 h) are compared. Scale bar represents 150 μm. (**B**) Quantification of the residual scratch area at the time point when the DMSO condition was completely closed by dividing the remaining scratch area by the initial scratch area in different HPV-negative and HPV-positive HNSCC cell lines. Statistically significant differences between IR and Mirin + IR were calculated with two-tailed Mann-Whitney test; no statistical test for Cal33; ** *p* = 0.0012. Each value represents mean ± SD (*n* ≥ 6)

Metastatic potential is generally relevant in cancer; for HNSCC especially lymph node metastases are prognostically relevant [37]. Consequently, a treatment that suppresses cancer cell spread would be highly beneficial. To analyze cell migration in the different treatment conditions, we used the scratch assay and recorded images of the scratch area every hour until the DMSO control was closed completely. Representative videos of the closing gap in every treatment condition for Cal33 and SBLF24 can be found in the supplementary information (compare Video 1, 3). In wells that were treated with Mirin (lower images) the gap closed later than in the DMSO control and in wells treated with IR. We chose the time point when the scratch in the DMSO control was closed completely for the analysis of each scratch assay experiment individually (compare to supplementary information_additional file 1, supplementary Table 1). We measured the remaining scratch area in the conditions at this time point and calculated the proportion of the remaining scratch area to the initial scratch area of 0.6 mm². As shown exemplarily for Cal33, the initial gap for the DMSO control and the combined treatment was the same at time point three hours, which was expected because it was directly after irradiation. In contrast, at time point 16 h, the cells closed the gap in the DMSO control completely while there was a remaining scratch in the combined condition, which we interpreted as a migration delay by Mirin + IR (Fig. 5, A). In HSC4, the residual scratch area was significantly (*p* = 0.0012) higher after the combined treatment compared to irradiation alone, which indicates a migration delay by Mirin. In all the other cell lines there were only tendencies detectable but no significant difference between irradiation alone and the combined treatment. In all cell lines except Cal33 and HSC4 the residual scratch area after the combined treatment was larger than with IR or Mirin alone, which indicates that the combined treatment reduced the migration ability of the cells the strongest. In the scratch assay there was no clear difference between the HPV-positive and HPV-negative cell lines. Notably, in Cal33 the scratch after IR was closed as fast as in the DMSO control. Consequently, no data are available and IR did not reduce the migration ability of this cell line. Cal33 was exceptional compared to the other cell lines as irradiation alone did not reduce their migration ability at all. Moreover, there was no difference between Mirin treatment and Mirin + IR, which is concomitant with HPV-negative radiation resistance (Fig. 5, B). For UD-SCC-2 and Detroit 562 no data acquisition was possible because the morphology of the cells did not allow the formation of a defined scratch. UD-SCC-2 were separated initially and did not form a closed cell layer in the inserts. Even if they started growing, after nine days - when the cells already died - the scratch area was not covered completely, which did not allow an analysis (compare to supplementary information_additional file 1, supplementary Fig. 2). Detroit 562 also did not form a proper cell layer and a clear scratch. Nevertheless, the reduction of their migration ability by Mirin was obvious (compare to supplementary information Video 2).

### Mirin in combination with IR elevates γH2AX level in HPV-negative HNSCCs

**Fig. 6:**
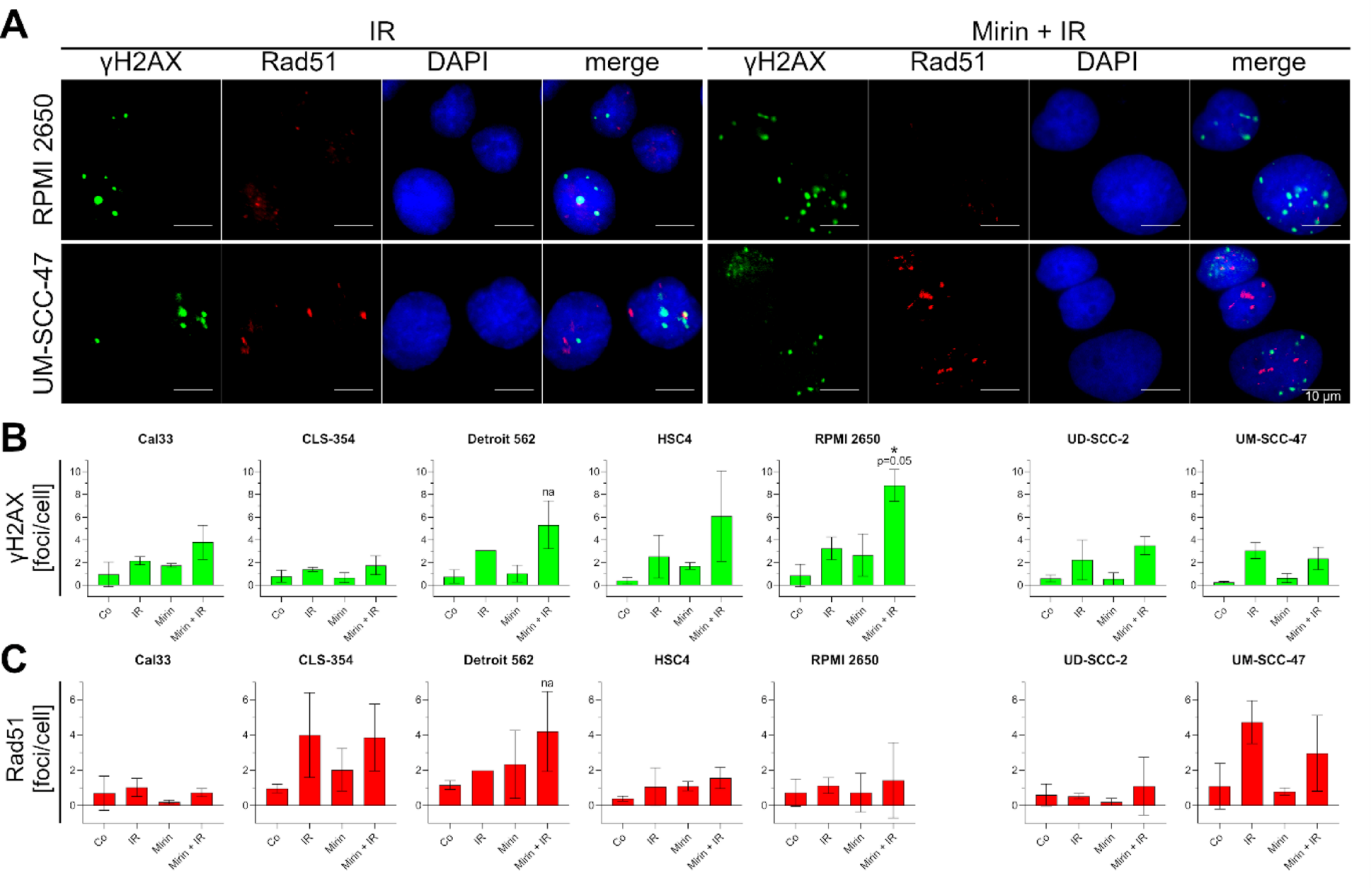
Determination of γH2A.X variant histone (H2AX) and Rad51 recombinase (Rad51) in HNSCC cell lines. (**A**) Representative images of immunostained RPMI 2650 and UM-SCC-47. Cells were treated with 30 µM Mirin and three hours afterwards additionally with a single dose of 2 Gy IR (Mirin + IR), only 30 µM Mirin (Mirin), or only 2 Gy IR (IR). Controls were completely untreated (Co). Exemplary IR and Mirin + IR are compared. γH2AX (green), Rad51 (red), and 2-(4-Amidinophenyl)−1*H*-indole-6-carboxamidine (DAPI) (blue) are presented separately and merged. Scale bar represents 10 μm. Quantification of γH2AX foci/cell (**B**) and Rad51 foci/cell (**C**) in different HPV-negative and HPV-positive HNSCC cell lines. Each value represents mean ± SD (*n* = 3; except HSC4, Co, *n* = 2; Detroit 562, IR, *n* = 1). Statistically significant differences between IR and Mirin + IR were calculated with one-tailed Mann-Whitney test; no statistical test (na: not applicable) for Detroit 562 due to limited repetitions

In the HNSCC cell lines we used immunostaining to detect DNA damage with γH2AX and HR activity with Rad51 remaining 21 h after irradiation. In a co-staining we marked γH2AX foci in the nucleus in green and Rad51 foci in red. We processed the representative images for better visibility of the foci; therefore, we reduced the background and intensified the colored signal. However, this was not applied for analysis. For γH2AX we counted every signal that was minimum ten grey scales lighter than the background as a focus, for Rad51 every signal that was at least 20 grey scales lighter (Fig. 6, A). In the HPV-positive UD-SCC-2 and UM-SCC-47 cell lines, only IR induced elevated γH2AX levels after 21 h, whereas neither Mirin nor the combined treatment did so compared to control or IR respectively. In contrast, the number of residual γH2AX foci per cell in RPMI 2650 was significantly increased in Mirin + IR compared to IR. In the other HPV-negative cell lines, the level of γH2AX foci/cell after combined treatment was notably higher than with IR alone, even if it was not statistically significant (Fig. 6, B). Rad51 foci/cell 21 h after irradiation did not change significantly when IR and Mirin + IR are compared. However, there seem to be two groups reacting differently to the treatment. In Cal33, HSC4, RPMI 2650, and UD-SCC-2 the Rad51 foci/cell were constant among all conditions. In CLS-354, Detroit 562, and UM-SCC-47 after irradiation and the combined treatment, Rad51 foci were elevated compared to control (Fig. 6, C). For every biological replicate and condition, all cell lines were seeded and stained on one eight-well chamber glass slide, which makes a repetition on another slide hardly comparable. Unfortunately, due to limited image quality, some images needed to be excluded from the analysis, which reduces the number of biological replicates for Detroit 562 and HSC4 (see also figure legend). This is why the results for those cell lines may only be interpreted as tendencies.

### Mirin does not influence phosphorylation sites of γH2AX, ATM Ser1981, p53 Ser15, and p53 Ser 20

**Fig. 7:**
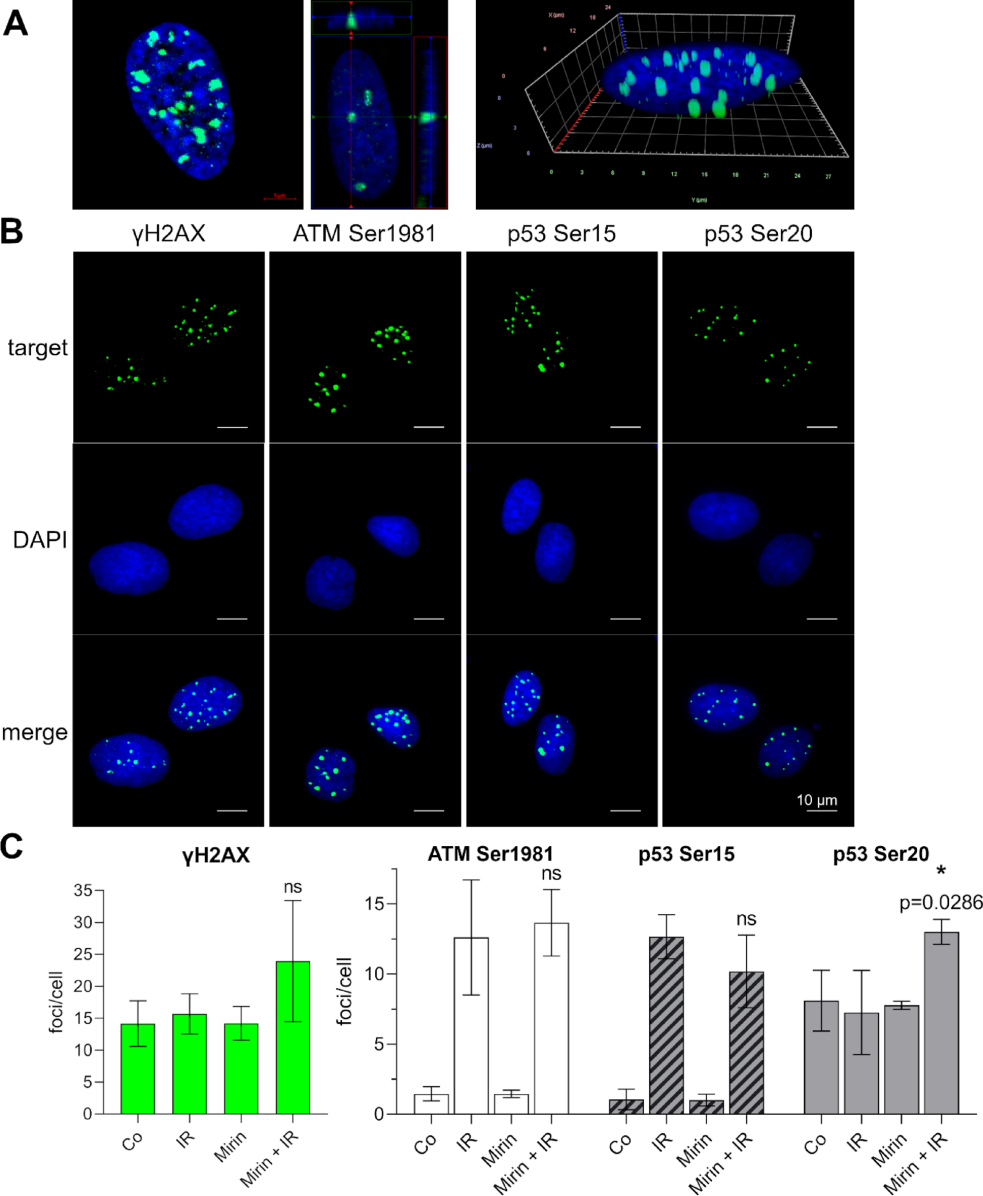
Phosphorylation of DNA repair proteins after Mirin treatment of fibroblasts. (**A**) Detailed representative images of γH2AX in SBLF24 treated with 2 Gy IR. Images were acquired by Zeiss, ApoTome.2 and Arivis software, interval of z-stacks was 0.24 μm. (**B**) Representative images of fibroblasts (SBLF24) treated with 30 µM Mirin and additionally with 2 Gy IR three hours afterwards (Mirin + IR). γH2AX and tumor protein P53 (p53) serine (Ser)20 were stained 21 h after irradiation. p53 Ser15 and ATM Ser1981 were stained three hours after irradiation. Presented are the target proteins in green, nuclei in blue (DAPI) and merged images. Scale bar represents 10 μm. (**C**) Quantification of γH2AX, ATM Ser1981, p53 Ser15, and p53 Ser20 foci/nucleus. SBLF24 were treated with 30 µM Mirin and three hours afterwards additionally with a single dose of 2 Gy IR (Mirin + IR), only 30 µM Mirin (Mirin), or only 2 Gy IR (IR). Controls were completely untreated (Co). Statistically significant differences between IR and Mirin + IR were calculated with two-tailed Mann-Whitney test; ns: not significant. Each value represents mean ± SD (*n* = 4; except p53 Ser20, Mirin, *n* = 3; ATM Ser1981, IR, *n* = 3)

To get a more detailed insight into the mode of action of Mirin we measured the phosphorylation of γH2AX, ATM, and p53 after Mirin treatment in healthy fibroblasts because their DDR should not be impaired and therefore the influence of Mirin should be detectable. We marked the targets with green fluorescence and counted γH2AX and p53 Ser20 foci that were at least ten grey scales lighter than the background and p53 Ser15 and ATM Ser1981 foci that were minimum five grey scales lighter than the background to determine foci/cell for the single targets (Fig. 7, A and B). The γH2AX level of SBLF24 was constant in control, IR, and Mirin. Only in the combined treatment it was elevated but not in a significant manner. ATM Ser1981 and p53 Ser15 foci are elevated only by IR, but not by Mirin and also the combination with Mirin had no statistically significant additional effect on the IR-induced foci. p53 Ser20 foci were constant in the control, IR, and Mirin, but were significantly elevated by the combined treatment compared to IR alone (Fig. 7, C).

## Discussion

The aim of this study was to investigate whether HNSCC cell lines can be radiosensitized by inhibiting their DDR - in particular the MRN complex - with the SMI Mirin. Standard treatment of HNSCC patients is frequently limited due to therapy resistance resulting in bad outcome [5]; especially in the HPV-negative cohort that has a poor prognosis [2–4]. To overcome this lack, the combination of established treatment options with targeted therapies is promising [38], but firstly preclinical data are necessary to evaluate the benefit. Therefore, we treated HNSCC cell lines with Mirin and additionally induced DNA damage by IR. Even if the effects of the single treatment conditions were weak, our data suggest a difference in the Mirin response between HPV-positive and HPV-negative cell lines. The HPV-negative cell lines seem to be radiosensitized by Mirin, while HPV-positive ones were not. What must be taken into account in radiochemotherapy is that patients receive repeated doses of radiation therapy alongside chemotherapy. Often, this involves 25 to 38 fractions, meaning even minor changes can have a substantial cumulative impact.

### Mirin might be effective in HPV-negative HNSCCs regarding cell death, cell cycle distribution, clonogenicity, and DNA damage

Cell death analysis of the normal tissue fibroblasts SBLF24 revealed the expected slight toxicity of IR but no induction of cell death by Mirin or the combined treatment. As G2/M is the most radiosensitive phase of the cell cycle [39], the increase of SBLF24 in G2/M after the combined treatment compared to IR alone indicates a radiosensitizing effect of Mirin in healthy cells. Nevertheless, the increase was much smaller than in the HNSCCs. Also Dupré et al. reported the induction of a G2/M block by Mirin [21]. In fractionated treatment schemes, patients get repeating doses on a regular basis; therefore, arresting cells in G2/M phase is problematic for healthy tissue but beneficial for the treatment of cancer cells because they are arrested in their radiosensitive state before the next fraction [40]. In the colony formation assay, the SF of SBLF24 was slightly reduced by Mirin alone but it was less toxic for the fibroblasts than for the HPV-negative HNSCCs. The SF of SBLF24 was significantly lower after the combined treatment compared to irradiation. Nevertheless, the normalization showed at least no additional effect of the combined treatment. However, the migration capacity of SBLF24 was nearly unaffected by Mirin and there was only a small additional effect in the combined treatment by Mirin compared to IR alone. Also, the γH2AX level 21 h post-irradiation in SBLF24 was not affected by Mirin (compare to Figs. 2, 3, 4, 5 and 7). These findings suggest that further investigation evaluating the side effects of Mirin are necessary.

The behavior of HPV-positive HNSCCs was similar to that of the fibroblasts. IR induced cell death, G2/M arrest, and elevated the level of γH2AX foci 21 h after irradiation, but the combination with Mirin had no additional effect. Only in the colony formation assay, the clonogenicity of UD-SCC-2 was reduced by the combined treatment compared to IR alone. The HPV-negative HNSCC cell lines responded completely different. Even if the results were not always statistically significant and consistent in the different assays, a promising radiosensitizing effect of Mirin in combination with IR was detected regarding cell death, G2/M arrest, clonogenicity, and DNA damage (γH2AX). In the scratch assay the difference between HPV-positive and HPV-negative HNSCC cell lines was not that clear but the radiosensitizing trend of Mirin regarding the reduction of migration capacity was detectable in all HNSCC cell lines (compare to Figs. 2, 3, 4, 5 and 6). When Dupré et al. established Mirin, they determined the half-maximal inhibitory capacity (IC_50_) of Mirin regarding phosphorylation of H2AX as 66 µM to evaluate the ATM activity after Mirin treatment in a cell free system [21]. Moreover, at 50 µM and 100 µM Mirin they detected a G2 arrest in a HEK293-derived reporter cell line (TOSA4) [21]. We showed that already the lower concentration of 30 µM Mirin might be effective in HPV-negative HNSCCs. However, in most of the assays and HPV-negative cell lines, radiosensitizing results were only tendencies and not all the results were statistically significant. Therefore, we suggest that our study supports the hypothesis that Mirin probably radiosensitizes HPV-negative HNSCC cell lines but further investigation is needed, for example with a higher dose of Mirin, higher doses of IR, or fractionated irradiation. Moreover, in vivo experiments would be necessary, especially to evaluate Mirin’s effect on healthy tissue.

Nevertheless, we conclude that there might be a difference in the reaction to Mirin depending on the HPV-status of the cell lines. Due to the different genetic characteristics of different cell lines, it is very unusual for a SMI to be effective in all HPV-negative cell lines. We have not seen it in this way before [41–43]. This could indicate that Mirin acts where there are no common mutations in the repair system or cell cycle control, and thus functions universally. Due to the multiple functions of Mirin’s target, Mre11, and thus the MRN complex, it is difficult to postulate what the target is. At the same time, these binding sites seem to be not present in HPV-positive cells, suggesting the involvement of p53 or retinoblastoma, since these two proteins are inactivated in HPV-positive cells [44]. At least slight radiosensitization of HPV-negative HNSCCs was detectable in all five tested cell lines; although the intensity of the effect was cell line dependent. However, even small effects would potentiate in a setting with several fractions as used in the clinics.

### The exact mode of action of Mirin remains unclear

Cancer cells often accumulated mutations in DDR while cancerogenesis [38], especially in HR [45–47]. We utilized Rad51 as a marker for HR [36]. As the MRN complex is highly associated with the functionality of HR, we investigated the change of Rad51 foci after Mirin treatment in the tested HNSCC cell lines. For cell lines capable of HR, we would expect an increase of Rad51 foci after induction of DNA damage by irradiation, while the addition of Mirin might reduce their number due to inhibition of HR. Dobler et al. already showed the HR-status of some of our cell lines stating that Cal33, HSC4, and UD-SCC-2 are HR-deficient while UM-SCC-47 are HR-proficient [48]. Consistent with these findings, we also detected no increase of Rad51 foci after our treatment in Cal33, HSC4, RPMI 2650, and UD-SCC-2, which is a hint that those cells are HR-deficient. CLS-354, Detroit 562, and UM-SCC-47 in contrast showed increased level of Rad51 after irradiation; however, Mirin had no effect on the number of Rad51 foci in those cell lines. To deeply analyze the effect of Mirin on HR and Rad51 foci, further experiments would be necessary. It could be useful to evaluate the HR-status of the cell lines with other methods and to measure Rad51 activity at a different timepoint, as the maximum of Rad51 foci can be detected about four hours after DNA damage [49] instead of 21 h as in our case. Currently, no reliable conclusion about the correlation between Rad51 expression and Mirin response can be done.

Further, we wanted to evaluate which DDR proteins mediate Mirin’s effect. To make sure that different mutational patterns of the cancer cell lines do not influence the results, we focused on the healthy fibroblasts. ATM is phosphorylated - among other phosphorylation sites – at Ser1981 after application of IR, which is crucial for the activation of ATM and eventually downstream DDR [50]. We detected ATM phosphorylation at Ser1981 after IR but Mirin did not impair this response. In contrast to our findings, Dupré et al. reported the inhibition of ATM phosphorylation at Ser1981 after DNA DSBs by Mirin; notably, these results were generated in cell free extracts in contrast to our mammalian cell culture model and a much higher concentration of 100 µM Mirin was used [21]. Moreover, they also had evidence that ATM activation does not rely on MRN only but can be activated independent from MRN when the amount of DNA damage is high [21].

p53 in general is a key protein in ensuring genome stability; we focused on the two phosphorylation sites Ser15 and Ser20 to control where Mirin impairs signaling pathways after DNA damage [51]. p53 Ser15 was phosphorylated after IR but Mirin did not reduce phosphorylation. In contrast, phosphorylation at p53 Ser20 was elevated only in the combined treatment.

Consequently, neither phosphorylation of ATM Ser1981, p53 Ser15, nor p53 Ser20 was inhibited by Mirin treatment so those are not the mediators of MRN inhibition by Mirin. As Mirin probably not only interacts with Mre11 but also other cellular functions [23], the definition of its exact mode of action is difficult. It remains unclear which proteins in the pathways downstream to ATM are responsible for Mirin’s effect and especially for the differences between HPV-positive and HPV-negative HNSCC cell lines.

HPV-positive and HPV-negative HNSCCs differ in their risk factors, tumor biology, DDR, and finally in their clinical outcome [6]. Regarding DDR, HPV-negative HNSCCs - mainly induced by tobacco or alcohol abuse – are distinguished by permanent DNA adducts whereas in HPV-induced HNSCCs DDR pathways are impaired by viral proteins [6]. DNA adducts can be repaired for example by base excision repair and nucleotide excision repair [6]. In HPV-positive HNSCCs the viral oncoproteins E6 and E7 are relevant for disturbing DDR by inhibiting p53 and retinoblastoma and therefore avoiding cell cycle arrest necessary for correct DNA repair and apoptosis resulting in genome instability and tumor progression [6, 52]. DDR can also be altered by mutations in p53 [53]; we previously reported the p53-status of our line panel. From the used HPV-negative cell lines, only RPMI 2650 is p53 wildtype, the status of CLS-354 is unknown, and the other cell lines (Cal33, Detroit 562, HSC4) are p53 mutated [43]. In contrast, HPV-positive HNSCCs are usually considered as p53 wildtype [54].

Consequently, the DDR mechanisms of HNSCC cell lines are highly dependent on the cells’ HPV-status. Dok et al. described that the inhibition of base excision repair radiosensitized HPV-positive HNSCC cells, while the inhibition of NHEJ and mismatch repair was beneficial independently from the HPV-status [55]. A study with radioresistant clones by Bamps et al. showed that HPV-positives ones responded with increased DDR to irradiation while HPV-negative ones with elevated replicative capacity [56]. Therefore, we suppose that the reaction to the Mirin treatment correlates with different alterations in DDR depending on the HPV-status of the cells.

Generally speaking, viral E6 and E7 cause a less efficient DDR in the HPV-positive HNSCCs compared to HPV-negative ones [6, 52]. Probably, the potential for Mirin inhibition is larger in HPV-negative HNSCCs because HPV-positive ones can intrinsically repair worse due to the viral oncoproteins. Another aspect potentially influencing the different reaction of HPV-positive and HPV-negative cell lines is that cancer cells, which often have impaired DDR, use alternative pathways besides HR and NHEJ for repairing DNA DSBs, for example base excision repair [38]. Hrudka et al. described an association between the tumor mutational burden and HPV-negativity [57]. Probably, the higher number of mutations in HPV-negative cell lines avoid the use of alternative DDR backup systems, which results in a disadvantage compared to HPV-positive cell lines. Alblihy et al. tested Mirin in BRCA2-deficient ovarian cancer cells and detected increased sensitivity compared to BRCA2-proficient cells regarding DSB, cell cycle arrest, and cell death. Moreover, they showed that Mirin-resistance correlates with increased DNA repair and reduced tumor protein P53 binding protein 1 activity [24]. The detailed underlying mechanism of Mirin’s effect in HPV-positive and HPV-negative HNSCC cell lines is not yet known but needs to be investigated further.

We generated first data that suggest a potential difference in Mirin response depending on the HPV-status of the treated HNSCC cells. However, there are already other targeted therapies described for HNSCCs that are specific for HPV-positive or HPV-negative cases. For example, the relevance of transforming growth factor-β (TGFβ) signaling in HPV-positive HNSCCs is known; TGFβ is relevant for different DNA repair pathways and its inhibition increases therapy response to DNA-damaging agents [58]. Liu et al. stated that TGFβ signaling is suppressed by HPV, which might be the reason for the better prognosis of HPV-positive HNSCCs [58]. Therefore, a TGFβ inhibition might be useful for other cancer types with sufficient TGFβ signaling [58]. Consequently, inhibiting TGFβ in HPV-negative HNSCC cell lines increased their response to IR treatment and other DNA-damaging agents, which shows the potential of this strategy to specifically sensitize HPV-negative HNSCC cells to achieve a comparable output as in HPV-positive ones [58, 59]. Another example are alterations in phosphatidylinositol 3-kinase (PI3K), which are associated with HPV-positive HNSCCs [60]. The majority of HNSCC patients has mutations in the PI3K/AKT/mTOR pathway; however, there is a distinct difference between HPV-positive and -negative cases. The prevalence of mutations in the PI3KCA gene is higher in HPV-positive cancers than in the negative ones and targeting this pathway seem to be especially promising in HPV-positive patients [61, 62]. Clinical trials investigating if this pathway is a useful target for HNSCC therapy and the corresponding influence of the HPV-status are currently ongoing [62]. Another type of targeted therapy is vaccination; besides the prophylactic HPV vaccine there are also therapeutic vaccines under evaluation that specifically target HPV-positive malignant cells [63]. These are just some examples of pathways that are under evaluation as potential targets for the treatment of HNSCCs taking into account their HPV-status. However, little is known about how to exploit the genetic differences for therapy and gaining further knowledge is necessary for improving therapy [61].

Our data suggest that Mirin shows the inclination to specifically radiosensitize HPV-negative HNSCCs, which is beneficial because they have the poorer prognosis than HPV-positive ones. However, the effect of Mirin was weak in some cell lines and assays and further validation is needed. The selected healthy control cell line SBLF24 showed good tolerance of Mirin except for cell cycle distribution and clonogenicity.

## Conclusions

We showed that HPV-positive HNSCCs would probably not benefit from Mirin, while Mirin might have radiosensitizing effects in HPV-negative HNSCCs that have a poor prognosis already. We also tested the treatment in healthy fibroblasts and can conclude that the side effects on healthy tissue might be low. The exact mode of action leading to the clearly different response in HPV-positive and -negative HNSCC cell lines remain unclear and need to be investigated further.

## Supplementary Information

Supplementary Material 1: Supplementary information_Video 1 Representative scratch assay observation of Cal33.mp4.

Supplementary Material 2: Supplementary information_Video 2 Representative scratch assay observation of Detroit 562.mp4.

Supplementary Material 3: Supplementary information_Video 3 Representative scratch assay observation of SBLF24..mp4.

Supplementary Material 4: Supplementary information_additional file 1.pdf.

## Data Availability

All data generated and presented during this study are available from the corresponding author on reasonable request.

## Abbreviations

APC: Allophycocyanin
ATM: Ataxia telangiectasia mutated
BRCA2: BRCA2 DNA repair associated
BSA: Bovine serum albumin
DAPI: 2-(4-Amidinophenyl)-1 H-indole-6-carboxamidine
DDR: DNA damage response
DMSO: Dimethyl sulfoxide
DNA: Deoxyribonucleic acid
DSB: Double strand break
DPBS: Dulbecco’s phosphate buffered saline
FBS: Fetal bovine serum
Gy: Gray
H2AX: H2A.X variant histone
HNSCC: Head and neck squamous cell carcinoma
HPV: Human papilloma virus
HR: Homologous recombination
IC50: Half-maximal inhibitory capacity
IR: Ionizing radiation
Mre11: MRE11 homolog, double strand break repair nuclease
MRN: Mre11-Rad50-Nbs1
Nbs1: Nibrin
NHEJ: Non-homologous end joining
PE: Plating efficiency
PI3K: Phosphatidylinositol 3-kinase
p53: Tumor protein P53
Rad50: Rad50 double strand break repair protein
Rad51: Rad51 recombinase
SD: Standard deviation
Ser: Serine
SF: Surviving fraction
SMI: Small molecule inhibitor
TBS: Tris-buffered saline
TGFβ: Transforming growth factor-β
7AAD: 7-Aminoactinomycin D
