# Supplementary material for: The inhibition of the MRN complex by Mirin radiosensitizes particularly HPV-negative HNSCC cell lines
Source: Cancer Cell Int. 2026 Feb 4;26:107. doi: 10.1186/s12935-026-04218-1 (PMC12934021; doi:10.1186/s12935-026-04218-1)
Supplement: Supplementary file 4 — Supplementary Material 4: Supplementary information_additional file 1.pdf. [file 12935_2026_4218_MOESM4_ESM.pdf]

Supplementary information

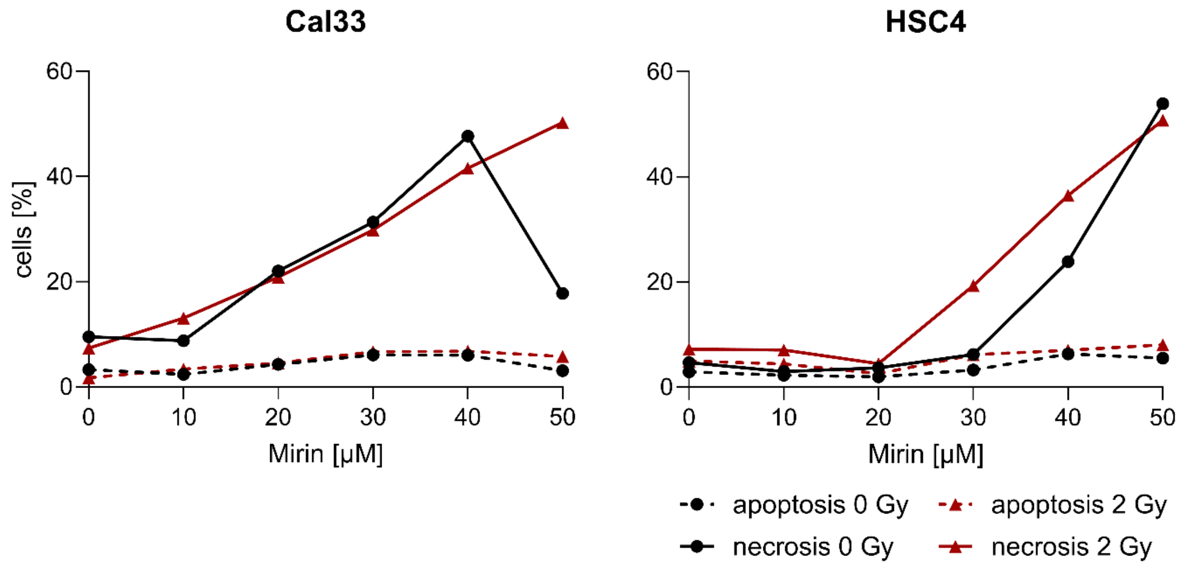

Supplementary Figure 1: Investigation of the toxicity of Mirin and determination of the Mirin dose to be used in further experiments based on the Cal33 and HSC4 cell lines. Cal33 and HSC4 were treated with 10 – 50 µM Mirin or the same volume DMSO as used for 50 µM (control, 0 µM Mirin) with or without 2 Gy IR. 30 µM of Mirin was determined to be suitable for the further experiments in this study. Analysis of apoptosis and necrosis by flow cytometry was carried out as described in the methods section.

Video 1: Representative scratch assay observation of Cal33. Upper left: DMSO control; upper right: IR; lower left: Mirin; lower right: Mirin + IR. Time stamp is represented as dd:hh:mm. 0 hours is directly after IR.

Video 2: Representative scratch assay observation of Detroit 562. Upper left: DMSO control; upper right: IR; lower left: Mirin; lower right: Mirin + IR. Time stamp is represented as dd:hh:mm. 0 hours is directly after IR.

Video 3: Representative scratch assay observation of SBLF24. Upper left: DMSO control; upper right: IR; lower left: Mirin; lower right: Mirin + IR. Time stamp is represented as dd:hh:mm. 0 hours is directly after IR.

Supplementary Table 1: The scratch assay was analyzed at the time point after Mirin treatment when the scratch in the DMSO control was completely closed. The table lists the used time point in hours for the individual cell lines and experiment repetitions (E).

Cell line	E 1	E 2	E 3	E 4	E 5	E 6	E 7	E 8	E 9
**Cal33**	18	16	10	14	11	10	17	12	n.a.
**CLS-354**	21	15	21	24	16	19	18	17	n.a.
**HSC4**	17	21	23	18	27	19	20	15	21
**RPMI 2650**	15	9	10	21	9	15	n.a.	n.a.	n.a.
**UM-SCC-47**	13	13	13	12	22	21	19	16	n.a.
**SBLF24**	110	87	54	68	98	76	n.a.	n.a.	n.a.

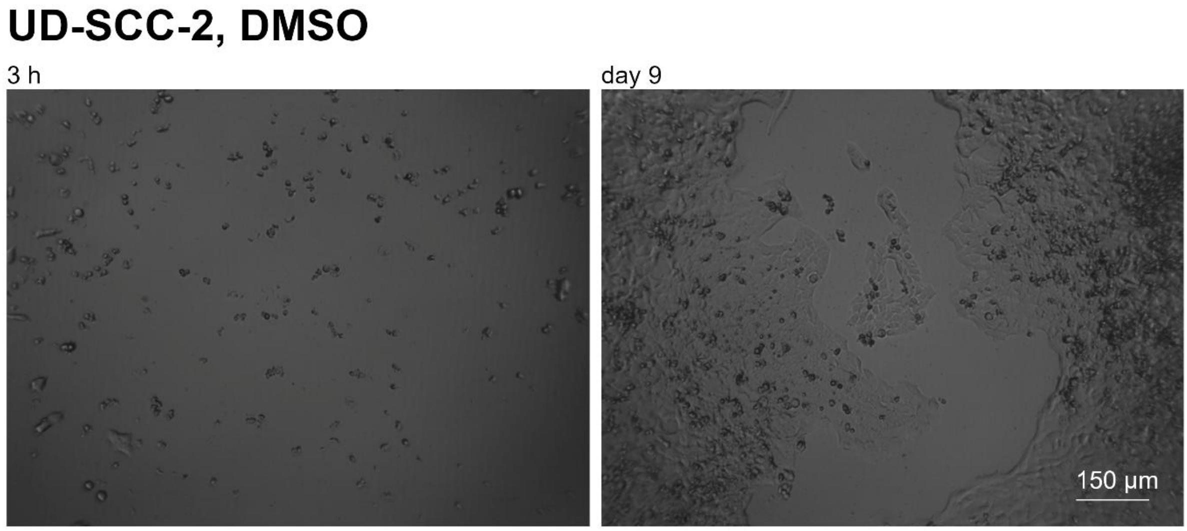

Supplementary Figure 2: Representative images of the scratch assay of UD-SCC-2. DMSO control of UD-SCC-2 three hours (initial image) and at day nine after Mirin treatment. Scale bar represents 150 µm. Because of the morphologic properties of this cell line there was no scratch in the initial image and even after nine days the scratch in the DMSO control was not closed completely. Therefore, a quantification of the scratch area in UD-SCC-2 was not possible.
